# Importance of Blood Glucose Measurement for Predicting the Prognosis of Long COVID: A Retrospective Study in Japan

**DOI:** 10.3390/jcm13144099

**Published:** 2024-07-13

**Authors:** Sho Yokoyama, Hiroyuki Honda, Yuki Otsuka, Kazuki Tokumasu, Yasuhiro Nakano, Yasue Sakurada, Yui Matsuda, Naruhiko Sunada, Toru Hasegawa, Ryosuke Takase, Daisuke Omura, Yoshiaki Soejima, Keigo Ueda, Masayuki Kishida, Fumio Otsuka

**Affiliations:** Department of General Medicine, Okayama University Graduate School of Medicine, Dentistry and Pharmaceutical Sciences, 2-5-1 Shikata-cho, Kita-ku, Okayama 700-8558, Japan; p5f11m3c@s.okayama-u.ac.jp (S.Y.);

**Keywords:** blood glucose, diabetes mellitus, long COVID, omicron variant, post-COVID-19 condition

## Abstract

**Purpose:** The present study aimed to clarify the effects of a hyperglycemic condition on the clinical consequences of long COVID. **Methods:** Among 643 patients who visited the outpatient clinic of our hospital from February 2021 to September 2023, long COVID patients were classified into a hyperglycemic (HG) group with casual blood glucose levels above 140 mg/dL and a normoglycemic (NG) group. The patients’ backgrounds, clinical symptoms, health status including the QOL evaluation scale (EQ-5D-5L), self-rating depression scale (SDS), and F-scale questionnaire (FSSG), blood test data, and recovery periods were analyzed. **Results:** The NG group included 607 patients with long COVID and the HG group included 36 patients with long COVID. Patients in the HG group were older than those in the NG group (55 vs. 41 years; *p* < 0.001) and included a larger percentage of males (67% vs. 44%; *p* = 0.009). The HG group had a larger percentage of patients with moderate-to-severe conditions in the acute infection phase (28% vs. 12%; *p* = 0.008), a higher BMI (25 vs. 22 kg/m^2^; *p* < 0.001), higher blood pressure (138/81 vs. 122/72 mmHg; *p* < 0.001), and a larger percentage of patients with an alcohol drinking habit (53% vs. 34%; *p* = 0.031). Long COVID symptoms and self-rated scales were not differed between the two groups; however, the laboratory data showed that liver and renal functions and metabolic data were significantly worse in the HG group. Although there was no apparent difference between the two groups in duration from the infection to the first visit, the HG group had a significantly longer period of recovery from long COVID (median period of 421 vs. 294 days; *p* = 0.019). **Conclusion:** A hyperglycemic state associated with other lifestyle-related diseases is associated with the prolongation of recovery from long COVID.

## 1. Introduction

The cumulative numbers of coronavirus disease 2019 (COVID-19) patients have been reported to be over 33 million in Japan and 770 million worldwide as of May 2024. Various variants with viral mutations have developed since the start of the COVID-19 pandemic in 2019 [1,2]. Approximately 10% to 30% of patients with COVID-19 have prolonged illness, including various physical and mental symptoms, after the process of the acute phase of COVID-19 [3,4,5,6].

Long COVID has been diagnosed as symptoms that occur within 2 to 3 months after infection with the SARS-CoV-2 virus and have continued for at least 2 months and cannot be explained by an alternative diagnosis [5]. The predominant symptoms of long COVID are general fatigue, so-called post-exertional malaise, and accompanying insomnia, headache, continuous cough, and dyspnea, and some patients also have cognitive dysfunction, myalgia, and slight fever [7,8]. The symptomatic characteristics of long COVID patients have gradually changed, and fatigue, headache, and insomnia became the main symptoms of the patients infected in the recent Omicron-dominant phase [9]. The number of long COVID patients with general fatigue has been constantly increasing in the Omicron-dominant phase regardless of the severity of the acute phase of infection [10]. The definition of long COVID among many fatigue-related diseases has become more difficult due to the necessity of differentiating it from various mimicking disorders.

Visceral fat has been shown to induce a certain level of chronic inflammation in patients with type 2 diabetes [11,12]. Chronic hyperglycemia and inflammation are also known to cause a decline in immunity by affecting blood sugar regulation and peripheral insulin sensitivity [13]. Diabetes is a poor prognostic factor for the acute-phase of COVID-19 and is known to increase the severity and mortality rate [14]. 

Since the proposed mechanisms underlying the pathophysiological aspects of long COVID include the reactivation of inactivated viruses or viral persistence, immunologic dysregulation or acquired autoimmunity, the dysregulation of microbiomes, abnormalities of the autonomous nervous system, tissue damage due to coagulation, and endocrine dysfunctions [7,15,16], the effects of a hyperglycemic condition might be involved in the mechanisms of the development and/or progression of long COVID conditions. In the present study, we focused on the significance of the casual blood glucose level, which can be easily measured, as a possible risk factor for long COVID, and we retrospectively examined its impact on pathological conditions and the treatment period. The aim of this study was to elucidate the impact of hyperglycemia on the clinical prognosis of long COVID patients.

## 2. Patients and Methods

### 2.1. Definitions of Patients’ Groups with Long COVID

In this study, a retrospective observational analysis at one hospital was conducted. We analyzed data for 643 patients who visited our COVID-19 aftercare clinic (CAC) at Okayama University Hospital from 22 February 2021 to 28 September 2023. We conducted detailed face-to-face interviews with all patients to assess their clinical symptoms of long COVID. Long COVID was defined as the persistence of symptoms for more than four weeks after the onset of COVID-19 [17], because our study included patients who were affected by COVID-19 before the current definition of long COVID. 

Based on casual blood glucose levels when they visited the CAC of our hospital, the long COVID patients were classified into two groups. Different from fasting blood glucose (FBS), for which the measurement requires a fasting period of more than 10 h from the preceding meal, casual blood glucose levels are blood sugar concentrations regardless of the time relationship between meals and the blood sampling time. Long COVID patients with casual blood glucose levels less than 140 mg/dL were defined as a normoglycemic (NG) group, and patients with casual blood sugar levels of 140 mg/dL or higher were defined as a hyperglycemic (HG) group [18,19].

### 2.2. Evaluation of Patients’ Clinical Conditions

Of the 802 patients who were scheduled for outpatient consultation, 159 patients were excluded. The patients who were excluded included 4 patients under 10 years of age, 4 patients without full examinations, 51 patients without the detection of COVID-19 and a confirmed date for COVID-19, 10 patients who visited the CAC within 4 weeks after COVID-19, and 90 patients without data for casual blood glucose levels. The remaining 643 patients with long COVID were included in this study. Medication for treating various symptoms related to long COVID was prescribed by the judgment of individual doctors in charge as well as information sharing at a medical conference every week, and the treatments for long COVID included anti-symptomatic treatments and Kampo medicine (traditional Japanese medicine) to alleviate symptoms for general fatigue [20,21].

The patients’ clinical backgrounds, clinical symptoms, clinical courses, and various self-rating scales were reviewed in the patients’ records. Data for the Japanese version of the fatigue assessment scale (FAS) scoring [22], the Japanese version of Euro QOL 5-dimensions 5-levels (EQ-5D-5L) scoring [23], the frequency scale for the symptoms of GERD (FSSG) scoring [24], and the self-rating depression scale (SDS) scoring to assess the patient’s depressive status [25] were analyzed. Systolic and diastolic blood pressures were measured by an automated sphygmomanometer in a relaxed sitting position. The definition of the severity of the acute phase of COVID-19 was based on the Japanese criteria [26].

### 2.3. Laboratory Examinations

The selection of biochemical examinations for long COVID was considered individually by the physicians’ team to evaluate various diseases. The laboratory data included blood cell counts (white blood cells: WBCs and hemoglobin: Hb), various inflammatory indicators (C-reactive protein: CRP and ferritin), liver functions (aspartate aminotransferase: AST, alanine aminotransferase: ALT, and ɤ-glutamyl transpeptidase: GTP) and kidney-related functions (creatinine, estimated glomerular filtration rate: eGFR, and uric acid: UA), trace elements (iron: Fe and zinc: Zn), and glucose and lipids (triglycerides: TGs, high-density lipoprotein cholesterol: HDL-C, and low-density lipoprotein cholesterol: LDL-C). Blood sampling was performed in a relaxed sitting position around late-morning to early-afternoon time, and the laboratory data were obtained by using an auto-analyzer system in our central laboratory.

### 2.4. Statistical Analysis

EZR (Easy R), version 1.61, a graphical user interface for R, version 4.2.2 [27], was used for the analysis. Fisher’s exact test was applied for the categorical variables and the Mann–Whitney U test was applied for the normally distributed variables; * *p* < 0.05 indicated statistically significant differences.

### 2.5. Ethics

The study protocol was approved by the Okayama University Hospital Ethics Committee (No. 2105-030) and complied with the Declaration of Helsinki. Information about this study was posted on the hospital’s website. If a patient wanted to opt out, an opportunity was given at any time. Informed consent was not required, since all of the data underwent anonymization procedures.

## 3. Results

First, as shown in Table 1, all of the long COVID patients were classified into two groups (a normoglycemic (NG) group (607 cases; 94.4%) and a hyperglycemic (HG) group (36 cases; 5.6%)) based on casual blood glucose levels when they visited our CAC. The mean level of casual blood glucose was significantly higher in the HG group than in the NG group (178.9 ± 8.5 vs. 100.5 ± 0.5 mg/dL; * *p* < 0.001). The ratio of males in the HG group was higher than that in the NG group (66.7% vs. 44.0%; * *p* = 0.009). The median age of patients in the HG group was higher than that of patients in the NG group (55 vs. 41 years; * *p* < 0.001). The median body mass index (BMI) of patients in the HG group was higher than that of patients in the NG group (25.2 vs. 22.2 kg/m^2^; * *p* < 0.001) (Table 1). 

The median levels of systolic blood pressure (SBP) and diastolic blood pressure (DBP) were higher in the HG group than in the NG group (SBP: 138 vs. 122 mmHg; * *p* < 0.001, DBP: 81 vs. 72 mmHg; * *p* = 0.001). There was no significant difference in the percentage of patients with a smoking habit between the NG group and the HG group. The percentage of patients with an alcohol drinking habit was higher in the HG group than in the NG group (52.8% vs. 34.4%; * *p* = 0.031). With regard to the clinical condition in the acute phase of COVID-19, there was no significant difference in the rate of hospital admission between the NG group and the HG group, but the percentage of patients who needed O_2_ and/or steroid therapy in the acute phase of COVID-19 was higher in the HG group than in the NG group (19.4% vs. 7.7%; * *p* = 0.024). The proportion of patients with a mild condition in the acute phase of COVID-19 was lower in the HG group than in the NG group and the proportion of patients with a moderate-to-severe condition in the acute phase of COVID-19 was higher in the HG group than in the NG group. There was no significant difference between the two groups in the proportions of patients who were vaccinated less than twice and more than twice. 

**Figure 1:**
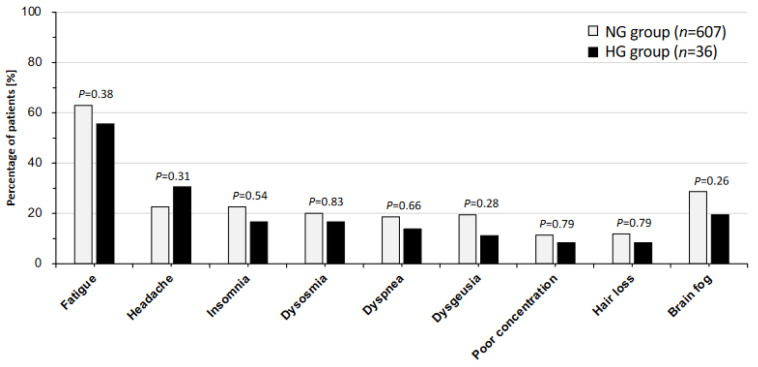
Percentages of patients with major symptoms of long COVID in the normoglycemic and hyperglycemic groups. The bar chart shows the percentages of patients with major symptoms of long COVID in the normoglycemic (NG) and hyperglycemic (HG) groups. The data were analyzed by using Fisher’s exact test; *p* < 0.05 indicates statistically significant differences.

**Figure 2:**
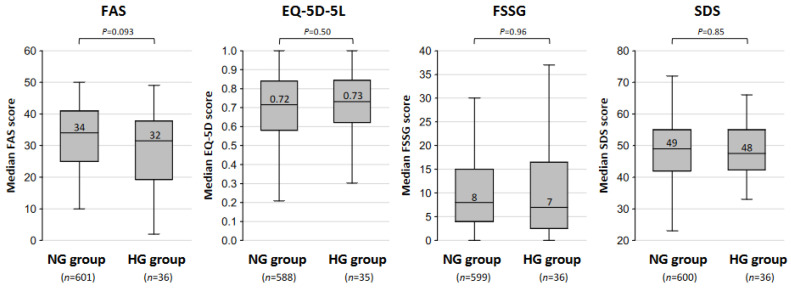
Self-rating scores in the normoglycemic and hyperglycemic groups. The medians and interquartile ranges of fatigue assessment scale (FAS) scores, Euro Qol 5-dimensions 5-levels (EQ-5D-5L) scores, frequency scale for the symptoms of GERD (FSSG) scores, and self-rating depression scale (SDS) scores in the normoglycemic (NG) and hyperglycemic (HG) groups are shown. The data were analyzed by using the Mann–Whitney U test; *p* < 0.05 indicates statistically significant differences.

The percentages of patients with each major long COVID symptoms in the NG group and the HG group are shown in Figure 1. The most frequent symptom was fatigue in both the NG group (63% of the patients) and HG group (56% of the patients). The percentages of patients with fatigue, insomnia (23% vs. 17%; *p* = 0.54), dysosmia (20% vs. 17%; *p* = 0.83), dyspnea (19% vs. 14%; *p* = 0.66), dysgeusia (19% vs. 11%; *p* = 0.28), poor concentration (11% vs. 8%; *p* = 0.79), hair loss (12% vs. 8%; *p* = 0.79), and brain fog (29% vs. 19%; *p* = 0.26) were higher in the NG group than in the HG group, although no significant differences were detected between the two groups. On the other hand, the percentage of patients with headache was lower in the NG group than in the HG group (23% vs. 31%; *p* = 0.31), though the difference was not statistically significant. The self-rating scores in the NG group and the HG group are shown in Figure 2. The median scores of the FAS (34 vs. 32 points; *p* = 0.093), EQ-5D-5L (0.72 vs. 0.73 points; *p* = 0.50), FSSG (8 vs. 7 points; *p* = 0.96), and SDS (49 vs. 48 points; *p* = 0.85) for the NG group and the HG group are shown. The scores of the FAS, EQ-5D-5L, FSSG, and SDS were not significantly different between the two groups.

**Figure 3:**
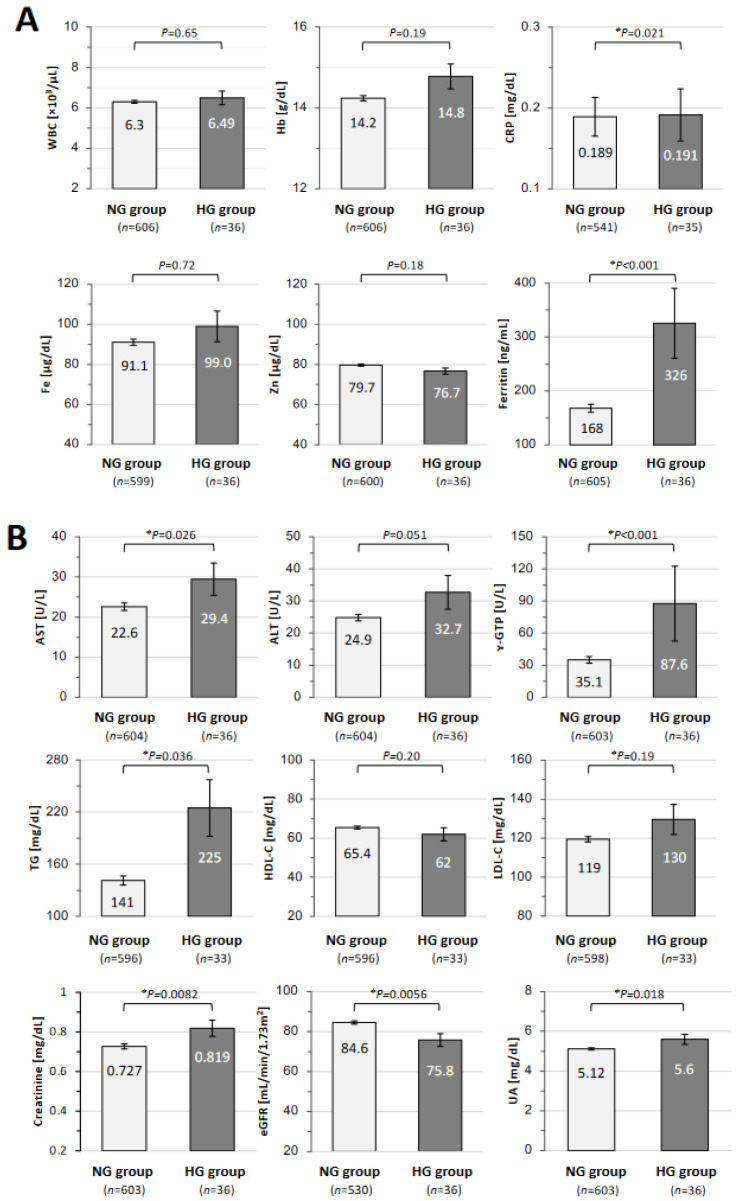
Differences in laboratory data in the normoglycemic and hyperglycemic groups. All of the laboratory data regarding (**A**) inflammatory and (**B**) metabolic findings are shown as means ± SEM in the normoglycemic (NG) and hyperglycemic (HG) groups. The data were analyzed by using the Mann–Whitney U test; * *p* < 0.05 indicate statistically significant differences. WBCs: white blood cells; Hb: hemoglobin; CRP: C-reactive protein; Fe: serum iron; Zn: zinc; AST: aspartate aminotransferase; ALT: alanine aminotransferase; ɤ-GTP: ɤ-glutamyl transpeptidase; eGFR: estimated glomerular filtration rate; TGs: triglycerides; HDL-C: high-density lipoprotein cholesterol; LDL-C: low-density lipoprotein cholesterol; UA: uric acid.

The results of the laboratory tests for blood cell counts, inflammatory markers, and trace elements in the NG group and the HG group are shown in Figure 3A. The mean level of C-reactive protein (CRP; 0.191 ± 0.032 vs. 0.189 ± 0.024 mg/dL; * *p* = 0.021) and the mean level of ferritin (326 ± 65 vs. 168 ± 7.4 ng/mL; * *p* < 0.001) were significantly higher in the HG group than in the NG group. There were no significant differences in the levels of WBCs, Hb, and serum Fe and Zn. The results of the laboratory tests for liver function, renal function, and lipids in the NG group and the HG group are shown in Figure 3B. The mean levels of serum AST (29.4 ± 4.0 vs. 22.6 ± 0.91 U/L; * *p* = 0.026), ɤ-GTP (87.6 ± 35 vs. 35.1 ± 3.1 U/L; * *p* < 0.001), TGs (225 ± 33 vs. 141 ± 5.3 mg/dL; * *p* = 0.0036), creatinine (0.819 ± 0.041 vs. 0.727 ± 0.013 mg/dL; * *p* = 0.0082), and UA (5.60 ± 0.25 vs. 5.12 ± 0.06 mg/dL; * *p* = 0.018) were significantly higher in the HG group than in the NG group. The mean value of eGFR (75.8 ± 3.2 vs. 84.6 ± 0.81 mL/min/1.73 m^2^; * *p* = 0.0056) was lower in the HG group than in the NG group. There were no significant differences between the two groups in the serum levels of ALT, HDL-C, and LDL-C.

**Figure 4:**
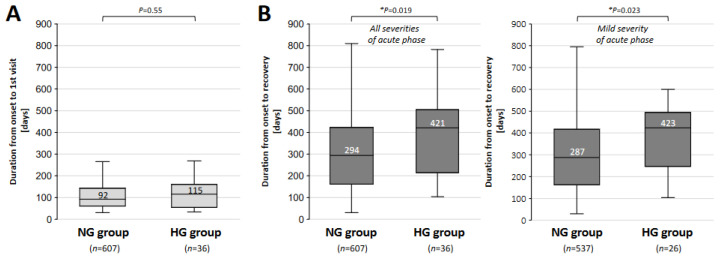
Comparison of the periods related to treatment for long COVID in the normoglycemic and hyperglycemic groups. (**A**) The medians and interquartile ranges of durations from onset to the first visit in the normoglycemic (NG) and hyperglycemic (HG) groups are shown. (**B**) The medians and interquartile ranges of durations from the onset of COVID-19 until recovery from long COVID for all patients (*left panel*) and for patients who had mild symptoms in the acute phase (*right panel*) in the normoglycemic (NG) and hyperglycemic (HG) groups. The data were analyzed by using the Mann–Whitney U test; * *p* < 0.05 indicates statistically significant difference.

The durations from the infection of the SARS-CoV-2 virus to the first visit to our CAC at Okayama University Hospital in the NG and HG groups are shown in Figure 4A. The median period was longer in the HG group than in the NG group (115 vs. 92 days; *p* = 0.55), though the difference was not significant. As shown in Figure 4B, the durations from the onset of COVID-19 until recovery from long COVID in all patients (*left panel*) and in patients who had mild symptoms in the acute phase of COVID-19 (*right panel*) were compared between the two groups. The median period for all patients was significantly longer in the HG group than in the NG group (421 vs. 294 days; * *p* = 0.019) (Figure 4B). The median period for patients who had mild symptoms in the acute phase of COVID-19 was also significantly longer in the HG group than in the NG group (423 vs. 287 days; * *p* = 0.023) (Figure 4B).

## 4. Discussion

In the present study, we focused on the hyperglycemic status of patients with long COVID. It was of interest that patients in the hyperglycemic group were older, included a larger percentage of males, included a larger percentage of patients with moderate-to-severe conditions in the acute phase of infection, and had obese and hypertensive characteristics. Of note, patients in the hyperglycemic group required a longer period for recovery from long COVID. Thus, a hyperglycemic state, in relation to the involvement of other lifestyle-related diseases, seems likely to affect the prognosis of long COVID.

In the general population, there is a correlation between BMI and the risk for the development of type 2 diabetes, and it has been shown that Japanese people have a high risk of developing diabetes even if their degree of obesity is low [28]. The rate of complication of hypertension in diabetic cases is approximately two times higher than that in non-diabetic cases, and the complication rate of diabetes in hypertensive patients is two to three times higher than that in individuals without hypertension [29]. The presence of diabetes is a poor prognostic factor for acute COVID-19, with higher rates of hospitalization and severe pneumonia than those in non-diabetic patients [30,31]. It has also been reported that hyperglycemia at hospital admission and a high level of hemoglobin A1c are significant predictors for death in type 2 diabetes patients with COVID-19 [32], and that hyperglycemic status caused by stress in diabetic patients hospitalized with COVID-19 is related to worse outcomes and in-hospital mortality [33]. The severity of the acute phase of COVID-19 is approximately two times higher and the mortality rate is two-and-a-half times higher in diabetic patients [34]. Here, we also found that the hyperglycemic group had a larger proportion of patients with severe COVID-19 such as cases requiring oxygen treatment or steroid treatment.

It has also been reported that the risk of developing long COVID is high in patients with diabetes [35]. In our previous study, a severe condition in the acute phase of COVID-19, as indicated by high anti-SARS-CoV-2 antibody titers in the patients’ sera, was shown to be involved in the sustained symptoms of long COVID [36]. Hence, long COVID tends to be prolonged in patients with severe disease in the acute phase of COVID-19 [36], which could be highly related to the hyperglycemic status.

Of interest, there was no difference between the two groups in the main symptoms of long COVID or the self-assessment scores (FAS, EQ-5D-5L, FSSG, SDS), but the period from infection to recovery from long COVID was significantly longer in the hyperglycemic group in our study. Regarding the QOL scores, it has been reported that EQ-5D-5L scores are generally lower in diabetic patients [37]. It has also been reported that there is generally a positive correlation between postprandial glucose levels and FSSG scores, although no correlation was observed between postprandial blood glucose levels and SDS scores [38,39]. Considering that SDS scores are also correlated with FSSG scores, there could be an inter-relationship between postprandial blood glucose levels and depressive status [38,39], which might be linked to the depressive mood seen in patients with long COVID. 

As for laboratory data, it has been reported that the serum ferritin level has a significant correlation with increases in blood glucose levels and is a possible risk factor for diabetes [40,41]. In this regard, our earlier study showed that serum ferritin levels were significantly increased in cases transitioning from long COVID to myalgic encephalomyelitis/chronic fatigue syndrome (ME/CFS), especially in female cases [42]. In the present study, the hyperglycemic group showed higher levels of serum inflammatory markers, including CRP and ferritin, than those in the normoglycemic group, in correlation with the patients’ background and being accompanied by increased levels of serum hepatic and lipid metabolism markers, including AST, ɤ-GTP, UA, and TGs. Non-alcoholic fatty liver disease is considered to have a bidirectional relationship with hyperglycemia and hyperinsulinemia, leading to de novo lipogenesis [43]. Since liver steatosis is also associated with insulin resistance [44], a hyperglycemic state and liver damage might mutually develop, leading to sustained conditions of long COVID.

Impairment of renal function and hyperuricemia were also observed in the hyperglycemic group in our study. Diabetic nephropathy not only causes a decline in renal function but also has a high risk of hyperuricemia, and hyperuricemia further increases the risk of developing diabetes [45]. Hyperinsulinemia promotes uric acid reabsorption by increasing the expression of the urate transporter of urate transporter 1 (URAT1) in renal proximal tubules, and it increases urine re-excretion in patients with decreased renal function, leading to a complication of hyperuricemia [46]. Systemic damage, including renal impairment, may also be involved in the prolongation of time for recovery from long COVID in patients with hyperglycemia.

Obesity and hyperlipidemia have been reported to be risk factors for patients who were diagnosed with hyperglycemia after contracting COVID-19, resulting in an increase in atherogenic index of plasma (AIP) values [47]. In addition, it has been shown that patients who complained of long COVID symptoms, even if they had mild symptoms during the acute phase of COVID-19, had a decrease in oxygen consumption accompanied by systemic oxygen supply disorder due to microcirculation dysfunctions [48]. Considering the present findings in this study, even if symptoms are mild during the acute phase of COVID-19, if the blood sugar level is high at any time, the symptoms of long COVID may be prolonged. It is therefore necessary to pay attention to prolongation of the period from onset to recovery in hyperglycemic patients with long COVID. 

The findings in the present study suggest that various factors such as inflammation and glucose metabolism are related to the long period for recovery from symptoms of long COVID in patients with hyperglycemia. As evidenced by the increases in serum levels of CRP and ferritin, high blood sugar levels may have led to sustained inflammation, the reactivation of inactivated viruses, and long-term latency of the SARS-CoV-2 virus. This pathogenesis may be related to the fact that, in cases of hyperglycemia, some other lifestyle factors such as liver function abnormalities, lipid abnormalities, and decreased renal function may also be complicated. Taken together, the results indicate that attention should be given to the correction and management of blood sugar levels for the prevention of long COVID. This prolongation of the healing period was the same even in mild cases during the acute phase of COVID-19. Although hyperglycemia was not directly involved in the differences in long COVID symptoms, it became clear that the symptoms were prolonged, accompanied by various changes in metabolism and inflammation.

The present retrospective study has various limitations. First, since this study was a retrospective study performed at a single institution in Japan, a prospective study is needed to accurately investigate the relationship between casual blood glucose levels and long COVID. Based on the statistics regarding the periods for treating long COVID between the normoglycemic and hyperglycemic groups, the statistical power was four tenths, suggesting that more sample sizes are required to conclude the present findings. We divided the patients into two groups in this study based on blood glucose levels only when the patients visited our CAC for the first time, but multiple blood glucose measurements would be required for accurate classification. In addition, we could not completely exclude the effects of steroid therapy during the acute phase of COVID-19 on hyperglycemia. Also, the treatments for long COVID patients were varied, but the prescriptions for the two groups were not completely adjusted. Second, patients who come to our hospital’s coronavirus aftercare outpatient clinic are only patients who are referred from various medical institutions, and we are dealing with a large number of patients who are seriously ill. However, the number of patients with moderate-to-severe symptoms in the acute phase of COVID-19 was small, and we need a large population for the accurate analysis of long COVID. Third, we could not analyze the details of the presence or absence of pre-existing diabetes or the related histories of diabetes treatment for all of the subjects. Therefore, we could not eliminate the possibility that casual blood glucose levels in this study were affected by pre-existing diabetes. In our future study, we need to examine hemoglobin A1c and conduct a glucose tolerance test to examine in detail whether the patients have impaired glucose tolerance or not. Fourth, we focused on patients who fulfilled the criteria for diagnosis of long COVID; however, we could not completely rule out the possibility that patients suffered from other diseases. Further examination of blood glucose levels and the risk of developing long COVID is required in a future study. Although no differences were observed in the clinical symptoms or self-assessment scores of long COVID patients with various blood glucose levels, further consideration based on the data from various facilities is required to determine the reason for the difference in periods from onset to recovery.

In conclusion, we uncovered that long COVID patients with hyperglycemia had included large percentages of patients with obesity, hypertension, and alcohol drinking habits, resulting in the prolonged periods until recovery from long COVID. This suggests the importance of intervening in underlying lifestyle-related diseases for improving the symptoms of long COVID, especially for elderly and male patients who tend to have a hyperglycemic condition.

## Figures and Tables

**Table 1:** Clinical backgrounds of long COVID patients with normal and high levels of glucose.

	NG Group *n* = 607 (94.4%)	HG Group *n* = 36 (5.6%)	*p*-Value
**Casual blood glucose** (mg/dL), mean ± SEM	100.5 ± 0.5	178.9 ± 8.5	* <0.001
**Patient’s profile**			
Male/Female (%)	267 (44.0)/340 (56.0)	24 (66.7)/12 (33.3)	* 0.009
Age, median [IQR]	41 [26, 50]	55 [48, 59]	* <0.001
BMI: median [IQR]	22.2 [20.3, 25.7]	25.2 [22.6, 30.2]	* <0.001
SBP: median [IQR]	122 [111, 135]	138 [119, 148]	* <0.001
DBP: median [IQR]	72 [65, 81]	81 [68, 93]	* 0.001
**Patients’ lifestyle**			
Smoking habit (%)	195 (32.4)s	14 (38.9)	0.465
Alcohol drinking (%)	207 (34.4)	19 (52.8)	* 0.031
**Clinical condition in acute phase of COVID-19**			
Hospital admission (%)	92 (15.2)	8 (22.2)	0.242
O_2_ and/or steroid therapy (%)	47 (7.7)	7 (19.4)	* 0.024
Mild condition (%)	537 (88.5)	26 (72.2)	* 0.008
Moderate-to-severe condition (%)	70 (11.5)	10 (27.8)	
**COVID-19 vaccination status**			
<2 doses (%)	240 (39.9)	9 (25.0)	0.081
≥2 doses (%)	361 (60.1)	27 (75.0)	

Long COVID patients were classified into a normoglycemic (NG) group with casual blood glucose levels less than 140 mg/dL and a hyperglycemic (HG) group with casual blood sugar levels of 140 mg/dL or higher. Data are shown as medians [IQR: interquartile ranges] and percentages (%), and were analyzed by using the Mann–Whitney U test or Fisher’s exact test; * *p* < 0.05 indicate statistically significant differences. BMI: body mass index; SBP: systolic blood pressure; DBP: diastolic blood pressure.

## Data Availability

Detailed data will be provided upon request to the corresponding author.

## Abbreviations

BMI: body mass index; COVID-19: coronavirus disease 2019; CAC: COVID-19 aftercare clinic; EQ-5D: Euro QOL five-dimensions five-levels scores; FAS: fatigue assessment scale; HG: hyperglycemic; NG: normoglycemic; PCC: post-COVID-19 condition, QOL: quality of life; SDS: self-rating depression scale; VAS: visual analog scale.
